# Retinal fluid is associated with cytokines of aqueous humor in age-related macular degeneration using automatic 3-dimensional quantification

**DOI:** 10.3389/fcell.2023.1157497

**Published:** 2023-03-08

**Authors:** Siyuan Song, Kai Jin, Shuai Wang, Ce Yang, Jingxin Zhou, Zhiqing Chen, Juan Ye

**Affiliations:** ^1^ Eye Center, The Second Affiliated Hospital, School of Medicine, Zhejiang University, Hangzhou, China; ^2^ School of Mechanical, Electrical and Information Engineering, Shandong University, Weihai, China; ^3^ School of Cyberspace, Hangzhou Dianzi University, Hangzhou, China

**Keywords:** age-related macular degeneration, cytokine, deep learning, optical coherence tomography, quantitative analysis, retinal fluid

## Abstract

**Background:** To explain the biological role of cytokines in the eye and the possible role of cytokines in the pathogenesis of neovascular age-related macular degeneration (nAMD) by comparing the correlation between cytokine of aqueous humor concentration and optical coherence tomography (OCT) retinal fluid.

**Methods:** Spectral-domain OCT (SD-OCT) images and aqueous humor samples were collected from 20 nAMD patient’s three clinical visits. Retinal fluid volume in OCT was automatically quantified using deep learning--Deeplabv3+. Eighteen cytokines were detected in aqueous humor using the Luminex technology. OCT fluid volume measurements were correlated with changes in aqueous humor cytokine levels using Pearson’s correlation coefficient (PCC).

**Results:** The patients with intraretinal fluid (IRF) showed significantly lower levels of cytokines, such as C-X-C motif chemokine ligand 2 (CXCL2) (*p* = 0.03) and CXCL11 (*p* = 0.009), compared with the patients without IRF. And the IRF volume was negatively correlated with CXCL2 (*r* = −0.407, *p* = 0.048) and CXCL11 (*r* = −0.410, *p* = 0.046) concentration in the patients with IRF. Meanwhile, the subretinal fluid (SRF) volume was positively correlated with vascular endothelial growth factor (VEGF) concentration (r = 0.299, *p* = 0.027) and negatively correlated with interleukin (IL)-36β concentration (*r* = −0.295, *p* = 0.029) in the patients with SRF.

**Conclusion:** Decreased level of VEGF was associated with decreased OCT-based retinal fluid volume in nAMD patients, while increased levels of CXCL2, CXCL11, and IL-36β were associated with decreased OCT-based retinal fluid volume in nAMD patients, which may suggest a role for inflammatory cytokines in retinal morphological changes and pathogenesis of nAMD patients.

## Introduction

As the aging of population in many countries, age-related degenerative diseases pose significant socio-economic challenges. One of the major degenerative diseases affecting the quality of life is age-related macular degeneration (AMD), affecting 8.7% of the global population (Wong et al., 2014). AMD can be neovascular or non-neovascular (Mitchell et al., 2018), of which neovascular AMD (nAMD) can lead to dramatic visual loss due to the destruction of retinal structure in the macular area, and permanent blindness in severe cases (Lim et al., 2012). Chronic inflammation, lipid deposition and oxidative stress are closely related to AMD pathogenesis (Miller, 2013; Fleckenstein et al., 2021). However, the specific association between the retinal morphological changes of AMD and chronic inflammation is still unclear.

Since its introduction, optical coherence tomography (OCT) rapidly became a widely used imaging technique for the diagnosis and treatment of a range of eye diseases affecting the choroid and retina (Huang et al., 1991). OCT imaging is non-invasive and fast to perform, compared with fundus fluorescein angiography (FFA). Compared to traditional fundus photography, OCT imaging could provide 2-3 dimensional structural information about the presence or absence of fluid in the intraretinal, subretinal, and the space below pigment epithelial layer, which are considered proxies for leakage (Wilde et al., 2015). OCT based fluid analyses of intraretinal fluid (IRF), subretinal fluid (SRF), and pigment epithelial detachment (PED) have proven promising for predicting functional deficits in nAMD (Klimscha et al., 2017). Previous studies have shown that retinal fluid change is a reliable OCT biomarker of nAMD (Schmidt-Erfurth et al., 2020; von der Burchard et al., 2018). The identification of retinal fluid based on OCT was subjective and time-consuming, and depended on the experience level of the clinician or observer, lacking an automatic quantification tool.

The emergence of deep learning has filled the gap in OCT imaging interpretation of retinal fluid and nAMD disease surveillance (Jin & Ye, 2022). Many studies have used deep learning technology to automatically detect and quantify baseline features in OCT from patients with AMD, such as IRF and SRF(Schlegl et al., 2018; Moraes et al., 2021). Sophie Riedl et al. related retinal fluid volume to visual acuity and found that the reduction of fluid volume in the retina was associated with visual recovery (Riedl et al., 2022). Quantitative retinal fluid volume has been used in many studies to evaluate the efficacy of anti-VEGF therapy (Schmidt-Erfurth et al., 2020; Michl et al., 2022). The application prospect of deep learning in OCT fluid segmentation makes it possible to quantitatively analyze the association between retinal fluid volume and inflammation.

Abnormal regulation of ocular inflammatory process plays an important role in the pathogenesis of AMD. The primary treatment for nAMD remains anti-vascular endothelial growth factor (VEGF) intravitreal injection, as clinical trials have demonstrated highly effective and tolerable safety profiles (Gillies et al., 2020; Woo et al., 2021; Holekamp et al., 2022). Unfortunately, some patients didn’t respond well to anti-VEGF therapy (Braimah et al., 2018), which may suggest that other inflammatory cytokines play a role in nAMD (Takeda et al., 2009). Aqueous humor and vitreous fluid can more directly and accurately reflect the intraocular inflammation of nAMD than serum. Previous studies have shown that the concentrations of many cytokines, including VEGF, angiogenin, growth-regulated oncogene, interferon γ-inducible protein (IP)-10 and macrophage inflammatory protein-1β, increased in aqueous humor and vitreous fluids of nAMD patients compared with normal subjects (Ambati et al., 2013; Agrawal et al., 2019; Tan et al., 2020; Zhou et al., 2020). Studies have shown that intravitreal triamcinolone acetonide can improve visual acuity and fundus performance in patients with nAMD in the short term (Danis et al., 2000). Therefore, understanding the relationship between intraocular cytokines and retinal morphological structure is of great significance for explaining the pathogenesis of AMD and developing new therapeutic strategies. However, few studies have linked retinal fluid to intraocular cytokines (Joo et al., 2021). There has not been a direct quantitative analysis of the association between retinal fluid volume and cytokines.

We aimed to explain the biological role played by cytokines in the eyes, the association between intraocular cytokines and retinal morphological changes, and the possible role of inflammatory cytokines in the pathogenesis of nAMD by quantitatively analyze the association between cytokine concentrations of aqueous humor and retinal fluid volume based on OCT.

## Materials and methods

### Study design

This study was a cross-sectional study. The Ethical Committee of the Second Affiliated Hospital, Zhejiang University School of Medicine approved the collection and study of human aqueous humor and OCT images. The patient’s aqueous humor cytokine data were obtained from another study at our hospital (Chen et al., 2020; Chen et al., 2021). All patients were treated in accordance with the Declaration of Helsinki, and informed consent was obtained from all participants before study participation.

### Inclusion/exclusion criteria

20 eyes of 20 consecutive patients with nAMD, who visited the Eye Center at the Second Affiliated Hospital of Zhejiang University (Hangzhou, China) from June 2017 to November 2018, were included in this study. The clinical diagnosis of nAMD was performed by FFA.

The inclusion criteria for this study were as follows: 1) age above 50 years; 2) First treatment; 3) No complications of other eye diseases. Exclusion criteria were: 1) pathological myopia; 2) Treatment history of nAMD, including intravitreal drug injection, photodynamic therapy, and steroid therapy; 3) Previous intraocular surgery, except for cataract surgery (which, for nAMD patients, must have been performed at least 12 months before inclusion); 4) Active inflammation, diabetes, use of immunosuppressive drugs and glucocorticoids, and local and systemic malignancies were excluded from this study.

### OCT image collection and annotation

According to the date of three aqueous humor collections, the patients’ closest OCT examinations to that date were collected. The days of difference between the two dates was no more than 7 days. Therefore, three aqueous humor samples did not match OCT on the corresponding date. Finally, 57 sets of data matched aqueous humor cytokine data and OCT images were included in the study. All OCT images were taken by a spectral-domain OCT B-scan (RTVue XR, Optovue, Fremont, CA, United States) at 960 × 405 pixels. One OCT visit consisted of 18 B scan images taken with radiography. We hoped to accurately obtain the volume of retinal fluid in OCT images, so we needed to pursue the accuracy of prediction segmentation of the model, rather than the generalization of the model. Three images were randomly selected from each patient’s baseline OCT examination and manually annotated by three ophthalmologists. They are considered experienced and highly trained in OCT fluid identification. IRF and SRF were independently labeled by three ophthalmologists, and inconsistent labels were determined by a majority after discussion.

### Aqueous humor sample collection and cytokines measurement

All nAMD patients received intravitreal injection of 0.5 mg ranibizumab for three consecutive months. Aqueous humor samples were collected three times from each patient, at baseline (before the first injection), 1 month (before the second injection), and 2 months (before the third injection). Using Luminex technology (Bio-Rad, Waltham, MA, United States) on a Bio-Plex MAGPIX system, a total of twenty-eight cytokines were detected in aqueous humor by a multiplex cytokine assay Kit (Developed Systems, Minneapolis, MN, United States). Not all cytokines can be detected in every aqueous humor sample. Finally, eighteen cytokines that could be detected in specific numbers were included in the study. To avoid imprecision between trials, cytokines were measured in all patient samples in one trial. Sample concentrations were calculated using multiparameter standard curves for each cytokine.

### Automatic quantification of retinal fluid volume

**FIGURE 1:**
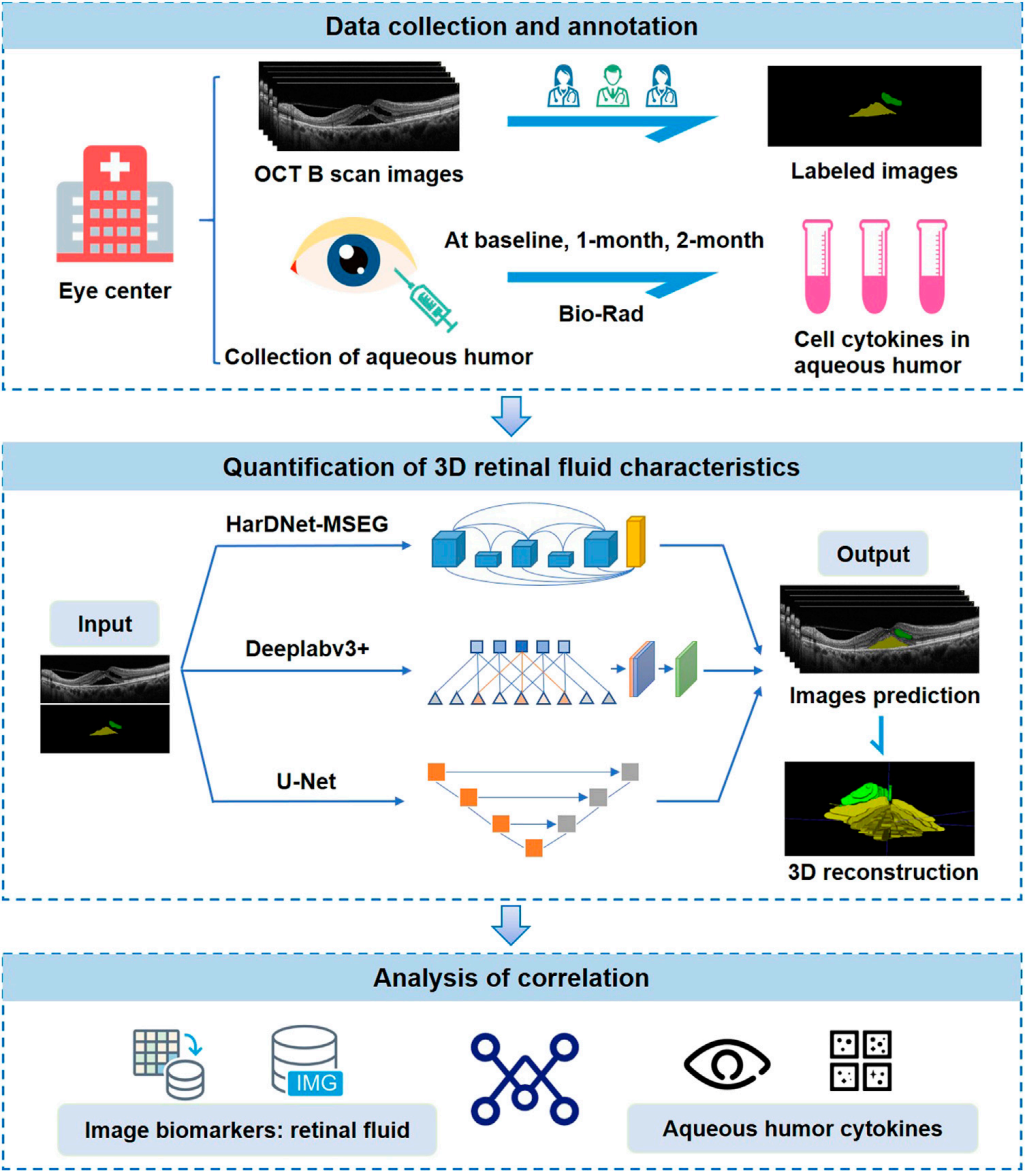
Study design. OCT images and aqueous humor samples are collected from eye centers and paired with each other. OCT images are annotated by three ophthalmologists. Retinal fluid volume is automatically segmented and quantified using three deep-learning models. Finally, the correlation between retinal fluid and aqueous humor cytokine levels is analyzed.

We selected three deep learning models that have been relatively successful in the field of fluid segmentation—HarDNet-MSEG (Huang, et al., 2021), Deeplabv3+(Chen et al., 2018) and U-Net (Ronneberger et al., 2015) -- to predict the retinal fluid. To train the model for retinal fluid segmentation, we first put 48,694 OCT images with 1,000 labeled images from Second Affiliated Hospital of Xi’an Jiaotong University (Xibei Hospital) into the network for transfer training. As shown in Figure 1, we put 1026 OCT images with 60 labeled images in this study into three deep learning models, and all three models output the predicted images. Then the Deeplabv3+ with the relatively good performance was selected from the three models as the quantitative analysis tool.

### 3D reconstruction and volumetric algorithm of fluid

To get the 3D segmentation result, we first placed the current 2D segmentation result to the corresponding position in the 3D space, and then used the 3D nearest neighbor interpolation to fill the unknown regions in the 3D space. Because the value of the segmentation result mask had a fixed range and wasn’t a continuous value, linear interpolation cannot be selected for the difference method, and the nearest neighbor interpolation was selected.

### Statistical analysis

All data were presented as either mean or mean ± standard deviation (SD). Pearson’s Correlation Coefficient (PCC) was used to quantify the correlation between the cytokine of aqueous humor and the OCT-based retinal fluid volume. Kolmogorov-Smirnov test was used to test the normal distribution of all continuous variables. Cytokine’s concentrations in the aqueous humor were compared between whether retina fluid presence or not using Student’s t-test. Due to the skewed distribution of the fluid volume, the fluid volume was calculated as log base 10 -- lg *(fluid volume)*.

SPSS software (version 25.0) was used for statistical analysis, *p* < 0.05 was considered statistically significant. Statistical maps were drawn by GraphPad Prism 8.

## Result

### Patient characteristics

A total of 57 sets of matched data from 20 eyes in 20 patients were finally included in the study. As shown in Table 1, the average of nAMD patient’s age was 66.65 ± 6.98 years (mean ± SD). Fourteen of the 20 nAMD cases (70%) were men, and nine of the 20 eyes (45%) were right eyes. And the biomarkers based on OCT were automatically measured by the deep learning model, and fluid volume was quantified. The average of IRF and SRF volume were 1.47×10^7^ ± 5.57 × 10^7^ μm^3^ and 3.39×10^7^ ± 5.16 × 10^7^ μm^3^ (mean ± SD). Due to the skewed distribution of the fluid volume, the fluid volume was calculated as log base 10. If the original fluid volume is 0, the logarithm cannot be taken, which was still regarded as 0. So the average of lgIRF (lgμm^3^) and lgSRF (lgμm^3^) were 2.54 ± 3.12 and 6.78 ± 1.52 (mean ± SD).

**TABLE 1:** Demographic and clinical characteristics of the study population.

Age (mean ± SD, years)	66.65 ± 6.98
Gender (n, %)	
Male	14 (70)
Female	6 (30)
Laterality of eye (n, %)	
OD	9 (45)
OS	11 (55)
OCT feature	
IRF volume (mean ± SD, μm^3^)	1.47×10^7^ ± 5.57×10^7^
lg (IRF volume)	2.54 ± 3.12
SRF volume (mean ± SD, μm^3^)	3.39×10^7^ ± 5.16×10^7^
lg (SRF volume)	6.78 ± 1.52
Total fluid volume	4.70×10^7^ ± 9.16×10^7^

IRF, intraretinal fluid; SRF, subretinal fluid.

### Deep learning model performance

The three model’s performance is shown in Table 2. The Deeplabv3+ showed the best performance for IRF segmentation, with the highest dice coefficient and precision of 0.802 and 0.952. The recall rate of Deeplabv3+ reached 0.794, which was not much different from the other two models. HarDNet-MSEG showed the best performance in SRF segmentation, with the dice coefficient and precision respectively reaching the highest values of 0.682 and 0.901. However, HarDNet-MSEG had poor performance in IRF segmentation, whose dice coefficient and precision were only 0.066 and 0.045. Meanwhile, Deeplabv3+ had the highest recall rate of 0.686 on segmental SRF. The dice coefficient and precision of Deeplabv3 + were 0.627 and 0.844, which were better than U-Net. Overall, the Deeplabv3+ model was selected as the tool for automated quantification of retinal fluid volume.

**TABLE 2:** Quantitative results for the performance of three models.

Method	IRF			SRF		
	Dice	Precision	Recall	Dice	Precision	Recall
HarDNet-MSEG	0.066	0.045	**0.886**	**0.682**	**0.901**	0.680
Deeplabv3+	**0.802**	**0.952**	0.794	0.627	0.844	0.686
U-Net	0.536	0.642	0.818	0.621	0.798	**0.736**

IRF, intraretinal fluid; SRF, subretinal fluid. That the bold values indicates, the best performance value of the three models in this vertical column of evaluation indicators.

### Comparison of intraocular cytokine between retinal fluid presence or absence

**FIGURE 2:**
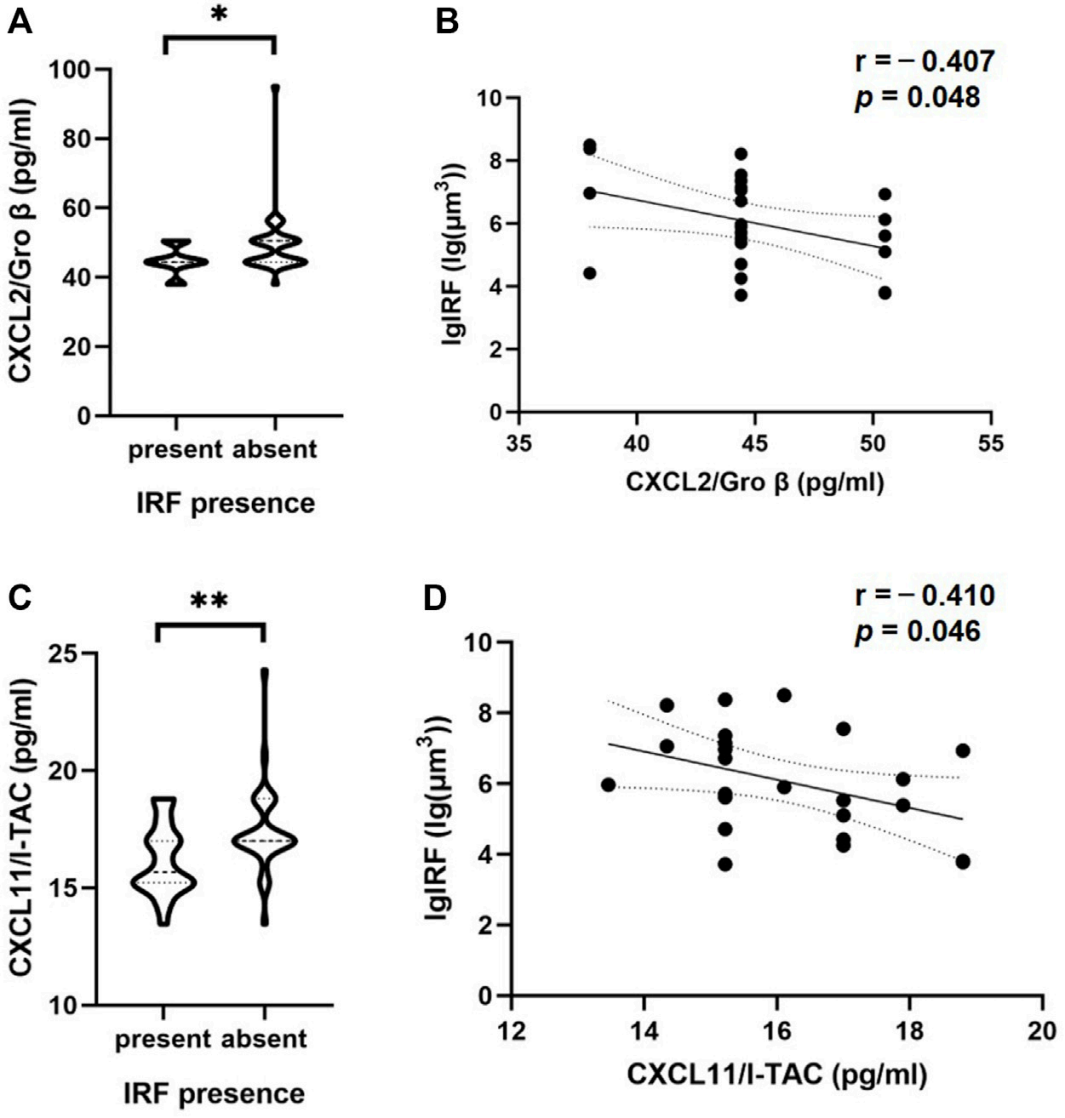
Correlations of cytokine in the aqueous humor and IRF based on OCT. The violin plot shows that there were significant differences between CXCL2/Gro β **(A)**, CXCL11/I-TAC **(C)** and the presence or absence of IRF. **(B, D)** In patients with IRF, there was a significant inverse correlation between these two cytokines and IRF volume based on Pearson’s correlation coefficient. Solid line, linear fitted trend line; Dotted line, 95% confidence interval. *p < 0.05, **p < 0.01.

Patients with nAMD were subdivided by OCT component on different dates. In the 57 OCT examinations, 24 were found to have IRF presence and 33 were found IRF absence, while 55 were found to have SRF presence and two were found SRF absence. The patients with IRF showed significantly lower levels of inflammatory cytokines, such as C-X-C motif chemokine ligand 2 (CXCL2) (*p* = 0.03) and CXCL11 (*p* = 0.009), compared with the patients without IRF (Table 3). Figures 2A, C shows the results of statistically significant intraocular cytokines in the presence or absence of IRF. This suggested that IRF was sensitive to change in intraocular cytokine concentration.

**TABLE 3:** Comparison of cytokine in the aqueous humor between patients with IRF or not and correlations between cytokine concentrations and IRF volume.

Cytokine in the aqueous humor (mean ± SD, pg/mL)	IRF			lg IRF (IRF presence)	
	Presence (n = 24)	Absence (n = 33)	*p*-value	Pearson’s correlation coefficient (95% CI)	*p*-value
IL-6	3.81 ± 3.74	5.02 ± 6.99	0.452	−0.073 (−0.463 to 0.341)	0.735
uPAR	69.23 ± 26.43	69.39 ± 16.04	0.979	−0.058 (−0.451 to 0.354)	0.790
CXCL10/IP-10/CRG-2	58.79 ± 66.47	115.40 ± 278.26	0.342	−0.175 (−0.541 to 0.246)	0.413
Endoglin	167.4 ± 15.20	173.12 ± 19.29	0.241	−0.086 (−0.473 to 0.329)	0.691
VEGF	33.68 ± 28.26	31.76 ± 21.82	0.777	0.038 (−0.371–0.435)	0.861
CXCL2/Gro β	44.87 ± 4.00	49.41 ± 9.21	**0.030**	−**0.407 (**-**0.696 to**-**0.004)**	**0.048**
CCL14/HCC-1	3,108.63 ± 1,109.56	3,154.48 ± 1,053.36	0.877	0.019 (−0.387–0.419)	0.929
CCL22/MDC	45.79 ± 2.60	46.70 ± 2.85	0.226	−0.152 (−0.523 to 0.268)	0.478
CCL21/6Ckine	21.29 ± 10.43	21.79 ± 8.32	0.843	0.110 (−0.307–0.491)	0.610
Thrombospondin-2	555.17 ± 161.62	524.02 ± 214.28	0.559	0.267 (−0.153–0.605)	0.207
CXCL11/I-TAC	16.19 ± 1.48	17.42 ± 1.79	**0.009**	−**0.410 (**-**0.698 to**-**0.008)**	**0.046**
Angiopoietin-1	78.88 ± 30.74	90.59 ± 43.90	0.276	0.062 (−0.350–0.454)	0.773
IL-36β/IL-1F8	1.26 ± 0.49	2.07 ± 4.10	0.345	−0.269 (−0.607 to 0.150)	0.203
HGF	328.69 ± 167.21	392.85 ± 392.80	0.463	0.009 (−0.396–0.411)	0.967
FGF acidic	33.45 ± 5.82	33.53 ± 7.33	0.967	−0.329 (−0.647 to 0.086)	0.117
PIGF	3.79 ± 1.21	3.35 ± 0.93	0.138	0.331 (−0.084–0.648)	0.114
ANGPTL4	5,178.5 ± 4,920.62	6,070.91 ± 5,702.11	0.547	0.008 (−0.397–0.410)	0.972
Endostatin	54,268.17 ± 16,879.46	56,772.09 ± 16,854.86	0.589	−0.109 (−0.491 to 0.308)	0.611

That the bold values indicates, *p* < 0.05 for *p*-values and Pearson’s coefficients.

However, we didn’t observe a statistically significant effect of the presence or absence of SRF on intraocular cytokine concentration (Table 4). Since the presence of SRF could not be identified in only two of the 57 OCT examinations, this statistical result may not be interpretive.

**TABLE 4:** Comparison of cytokine in the aqueous humor between patients with SRF or not and correlations between cytokine concentrations and SRF volume.

Cytokine in the aqueous humor (mean ± SD, pg/mL)	SRF			lg SRF (SRF presence)	
	Presence (n = 55)	Absence (n = 2)	*p*-value	Pearson’s correlation coefficient (95% CI)	*p*-value
IL-6	4.61 ± 5.95	1.61 ± 0.00	0.486	0.035 (−0.233–0.297)	0.803
uPAR	69.44 ± 21.41	66.07 ± 3.14	0.827	−0.059 (−0.319 to 0.209)	0.668
CXCL10/IP-10/CRG-2	93.99 ± 221.41	24.84 ± 12.02	0.666	0.057 (−0.212–0.317)	0.680
Endoglin	170.98 ± 18.17	163.40 ± 0.00	0.565	0.001 (−0.265–0.266)	0.996
VEGF	32.21 ± 25.03	42.55 ± 11.6	0.570	**0.299 (0.036 to 0.523)**	**0.027**
CXCL2/Gro β	47.50 ± 7.92	47.46 ± 3.05	0.995	0.011 (−0.255–0.276)	0.934
CCL14/HCC-1	3,150.38 ± 1,078.57	2,717.00 ± 961.00	0.584	0.150 (−0.120–0.399)	0.275
CCL22/MDC	46.39 ± 2.81	44.24 ± 0.00	0.291	−0.075 (−0.334 to 0.194)	0.585
CCL21/6Ckine	21.44 ± 9.34	25.29 ± 5.90	0.573	0.027 (−0.240–0.290)	0.848
Thrombospondin-2	537.80 ± 197.59	518.99 ± 61.28	0.895	0.048 (−0.221–0.309)	0.730
CXCL11/I-TAC	16.94 ± 1.79	15.67 ± 0.44	0.325	−0.011 (−0.275 to 0.256)	0.939
Angiopoietin-1	85.55 ± 39.89	88.52 ± 17.78	0.919	0.140 (−0.130–0.391)	0.307
IL-36β/IL-1F8	1.75 ± 3.22	1.16 ± 0.08	0.798	−**0.295 (**-**0.520 to**-**0.032)**	**0.029**
HGF	370.25 ± 324.44	244.46 ± 3.95	0.592	0.055 (−0.214–0.315)	0.692
FGF acidic	33.67 ± 6.79	28.67 ± 1.34	0.311	0.009 (−0.257–0.273)	0.951
PIGF	3.54 ± 1.09	3.26 ± 0.31	0.720	0.097 (−0.173–0.353)	0.482
ANGPTL4	5,773.27 ± 5,485.33	3,547.00 ± 582.00	0.575	−0.098 (−0.354 to 0.172)	0.478
Endostatin	55,843.29 ± 17,120.05	52,267.00 ± 8,803.00	0.774	0.090 (−0.179–0.347)	0.512

That the bold values indicates, *p* < 0.05 for *p*-values and Pearson’s coefficients.

### Associations between intraocular cytokines and retinal fluid volume based on OCT

To further investigate the associations between the volume of IRF/SRF and cytokines of aqueous humor, we performed Pearson’s coefficient correlation analysis in patients with IRF/SRF. Because of the skewed normal distribution of retinal fluid volume, we took logarithm of retinal fluid volume for analysis. Among the eighteen cytokines, the same two cytokines were significantly correlated with IRF volume in the patients with IRF (Table 3). CXCL2 and CXCL11 were statistically significantly correlated with IRF (*p* = 0.048 and *p* = 0.046). Moreover, the IRF volume was negatively correlated with CXCL2 (*r* = -−0.407, 95% CI: −0.696 to−0.004) and CXCL11 (*r* = −0.410, 95% CI: 0.698 to-0.008) concentration (Figures 2B, D).

**FIGURE 3:**
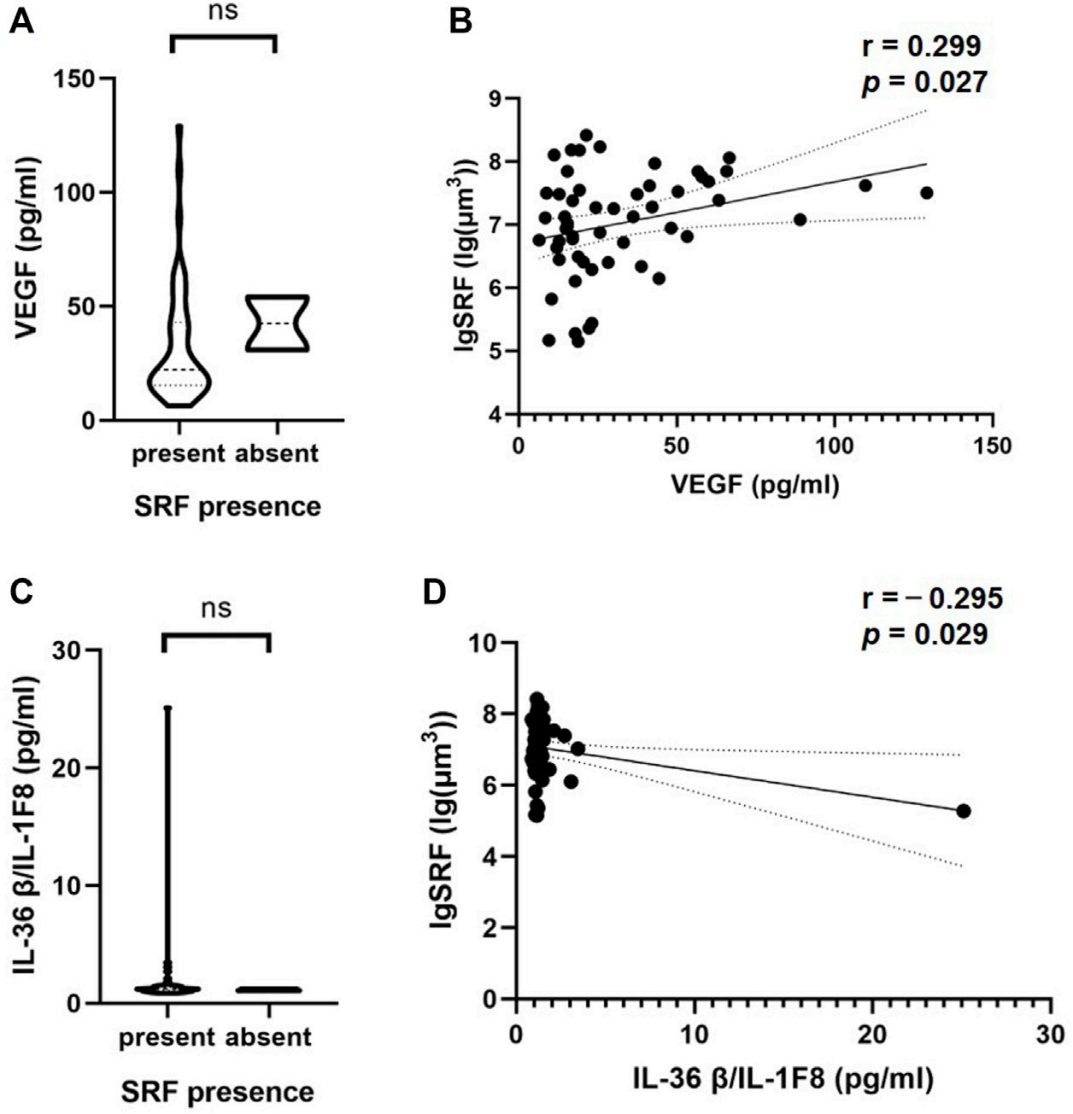
Correlations of cytokine in the aqueous humor and SRF based on OCT. The violin plot shows that there were no significant differences between VEGF **(A)**, IL-36β/IL-1F8 **(C)** and the presence or absence of SRF. **(B, D)** In patients with SRF, there was a significant inverse correlation between these two cytokines and SRF volume based on Pearson’s correlation coefficient. Solid line, linear fitted trend line; Dotted line, 95% confidence interval. *p < 0.05, **p < 0.01.

Interestingly, a correlation between aqueous humor cytokines and SRF volume was observed in nAMD patients with SRF (Table 4). VEGF and interleukin (IL)-36β were statistically significantly correlated with SRF volume (*p* = 0.027 and *p* = 0.029). Moreover, the SRF volume was positively correlated with VEGF concentration (*r* = 0.299, 95% CI: 0.036–0.523) and negatively correlated with IL-36β concentration (*r* = −0.295, 95% CI: −0.520 to−0.032) (Figures 3B, D). At the same time, we observed the classification of these two cytokines in patients with or without SRF (Figures 3A, C).

## Discussion

In this study, we investigated the association of OCT-based retinal fluid with various cytokines of aqueous humor. The use of artificial intelligence to automatically analyze OCT images and allow quantification of retinal fluid volume is gaining popularity worldwide. A large number of deep learning models have been developed to quantify fluid volume features in OCT, and the deep learning tool used in our study reported non-inferior model performance in the field of retinal fluid segmentation (Lu et al., 2019; Terry et al., 2021). In previous studies, the central macular thickness based on OCT was associated with cytokine levels by manual measurement (Joo et al., 2021). Joseph R Abraham et al. quantified retinal fluid volume using deep learning in diabetic retinopathy and correlated it with aqueous humor cytokines (Abraham et al., 2021). Two-dimensional images alone cannot accurately measure the characteristics of retinal fluid, so we used SD-OCT volume model to quantitatively analyze the volume of retinal fluid. Deep learning has significant advantages in quantitative analysis of retinal fluid volume, saving labor cost and time cost. For the first time, we used deep learning to quantify retinal fluid volume in AMD patients and quantitatively associated it with aqueous humor cytokine levels.

The cytokines of aqueous humor and vitreous fluid can better reflect the disease status of the retina than those in serum. Diagnostic sampling of vitreous fluids helps in the diagnosis and treatment of ocular diseases (Funatsu et al., 2006). Obtaining vitreous fluid from a patient’s eye is riskier than collecting aqueous humor, which can lead to side effects such as vitreous bleeding and retinal detachment. Significant correlations have been reported between cytokine levels in aqueous humor and vitreous fluids (Butler et al., 2020). In our study, cytokines of aqueous humor rather than serum were measured and associated with retinal fluid volume based on OCT.

The role of VEGF in AMD has been strongly supported in several studies (Lee et al., 2018; Yang et al., 2022). VEGF appears to be a major stimulator of neovascularization growth originating from the retinal and choroidal vasculature (Marneros, 2021). There have been many studies showing significant resolution of SRF after anti-VEGF treatment (Amoaku et al., 2015; Schmidt-Erfurth et al., 2020), suggesting that the reduction of VEGF was associated with SRF shrinkage. These results were consistent with our study, in which we found that decreased VEGF concentration of aqueous humor was associated with reduced SRF volume in nAMD patients with SRF. However, a few studies have reported non-resolution of SRF in the patients with AMD despite anti-VEGF therapy (Hosseini et al., 2021).

The mast cell and macrophage chemokine CXCL2 was found to control the early stages of neutrophil recruitment during tissue inflammation (De Filippo et al., 2013). Md Huzzatul Mursalin et al. found the utility of CXCL2 as a potential target for anti-inflammatory therapy for intraocular inflammation in mice (Mursalin et al., 2021). Retinal pigment epithelium dysfunction caused by abnormal inflammatory responses is associated with the pathogenesis of AMD (Ambati et al., 2013). Previous studies have reported that human retinal pigment epithelium cells produce CXCL11 under inflammatory conditions (Kutty et al., 2015). IL-36β protected mice from herpes virus infection and had a regulatory effect on immune function (Milora et al., 2017). Our study was the first to show that CXCL2 and CXCL11 concentration in aqueous humor of nAMD patients with IRF is significantly lower than that of patients without IRF. Meanwhile, in patients with IRF, the concentration of CXCL2 and CXCL11 was negatively associated with the volume of IRF based on OCT. We also found that the concentration of IL-36β was negatively associated with the volume of SRF based on OCT in patients with SRF. The reduction in IRF and SRF volume in some sense represents a reduction in the degree of disease activity. These suggested that chemokines may play a role in regulating the retinal inflammatory response and macrophage recruitment may partially prevent the deposition of harmful substances in the retina. Appropriate concentration of chemokines can recruit macrophages to gather in the eye, phagocytic harmful substances and reduce inflammatory exudation, so the volume of retinal effusion is reduced.

The pathological environment in the eye, such as ischemia, hypoxia or inflammation, is a pro-angiogenic factor that can lead to the formation of new blood vessels, and corresponding cytokines are involved in these processes. Research into the cytokines and retinal fluid volume associated with AMD pathogenesis may provide new insights into the development of targeted drugs and more effective AMD therapies.

Our study had certain limitations. The number of patients included in the study was only 20 patients, although each patient provided data from three pairs. This was limited by the invasive procedure of obtaining aqueous humor, which can be obtained only in patients receiving anti-VEGF therapy. At the same time, there were fewer data for the absence of SRF, which also led to the statistical results being not meaningful for interpretation.

In summary, changes in cytokine levels after treatment support the notion that the intraocular cytokines other than VEGF were involved in pathogenesis of AMD and morphological changes of retina. Our study suggested that CXCL2, CXCL11 and IL-36β might be some biomarkers or predictors of response to anti-VEGF therapy or corticosteroids, thereby allowing targeted and individualized therapy guided by cytokine levels.

## Data Availability

The raw data supporting the conclusion of this article will be made available by the authors, without undue reservation.
